# Home‐based collaborative management of bullous pemphigoid with delayed seropositive conversion of anti‐BP180‐NC16a antibody

**DOI:** 10.1002/jgf2.70002

**Published:** 2025-02-13

**Authors:** Hiromitsu Yamashita, Takayuki Ando, Hirohisa Fujikawa, Masayoshi Shiota

**Affiliations:** ^1^ Kawakita Family Clinic Minamiasagaya Tokyo Japan; ^2^ Center for General Medicine Education, School of Medicine Keio University Tokyo Japan; ^3^ Department of Medical Education Studies, International Research Center for Medical Education, Graduate School of Medicine The University of Tokyo Tokyo Japan

**Keywords:** bullous pemphigoid, collaborative practice, dermatology, home‐based care

## Abstract

We report the case of an 82‐year‐old male with a history of multiple cerebral infarctions who developed bullous pemphigoid while receiving home‐based care. Anti‐BP180‐NC16a antibody was initially negative, but later became positive as the disease severity worsened. The patient was successfully managed at home through a collaborative effort between a home‐visiting physician (general practitioner), dermatologist, nursing staff, and pharmacists. This case highlights the value of reevaluating anti‐BP180‐NC16a antibody levels and the feasibility of home‐based care for severe bullous pemphigoid in bedridden patients.

## Graphical abstract

We present the case of an elderly male with bullous pemphigoid, initially negative for the anti‐BP180‐NC16a antibody, which later became positive as disease activity progressed. The patient was successfully managed at home through multidisciplinary collaboration, including a general practitioner, dermatologist, nurses, and pharmacists.

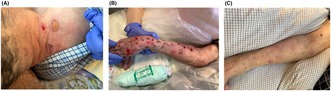

## 1 BACKGROUND

Bullous pemphigoid is an autoimmune disease commonly affecting elderly patients that is characterized by subepidermal blisters caused by autoantibodies against hemidesmosomal constituent proteins (BP180 and BP230).^1^, ^2^ This is typically managed by hospitalization and systemic corticosteroid therapy.^2^ Since false negatives may arise from measuring anti‐BP180‐NC16a antibody levels, with a sensitivity of 72%–89%, a skin biopsy is usually done for diagnosis.^1^ Although the anti‐BP180‐NC16a antibody reflects disease activity, delayed seropositivity with worsening symptoms has not been reported.^3^, ^4^ Herein, we present a case of bullous pemphigoid in a multimorbid, bedridden patient who initially tested negative for anti‐BP180‐NC16a antibody, which later turned positive with increasing disease activity. Despite the severity of the disease, collaborative home‐based care enabled its successful management at home.

## 2 CASE PRESENTATION

An 82‐year‐old male presented with tense blisters on the posterior neck (largest diameter: 10 mm), first identified by a home‐visiting nurse. The patient had multiple comorbidities, such as heart failure, seizure, and multiple cerebral infarctions, which necessitated a gastrostomy and limited his verbal communication. He required full assistance with activities of daily living (ADLs) and received home‐based medical care. His current medications included edoxaban, vonoprazan, valproic acid, and risperidone.

**FIGURE 1:**
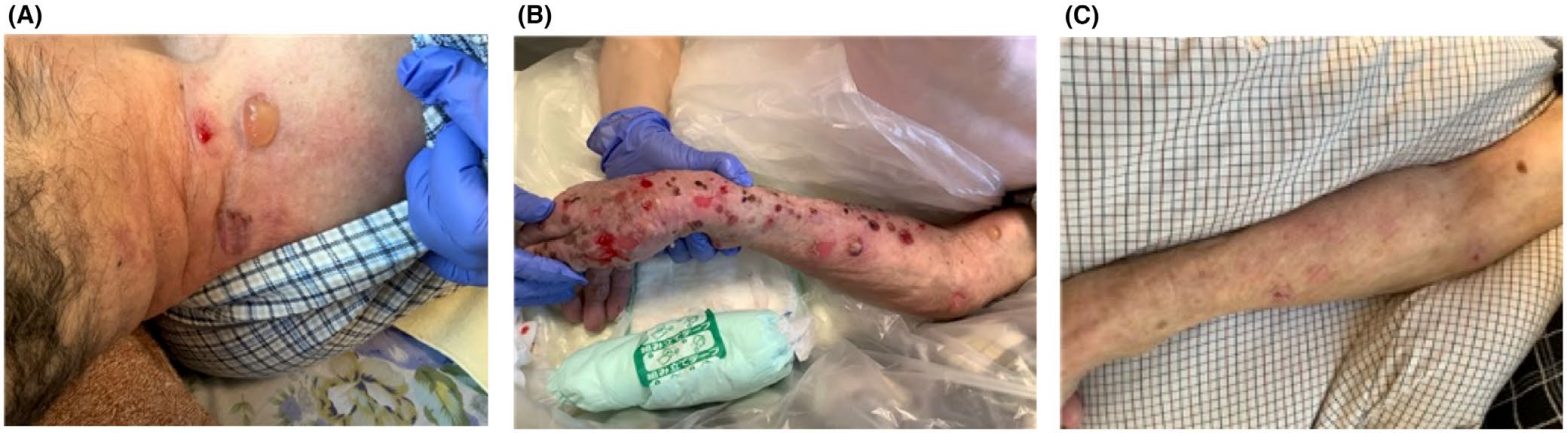
Clinical images of the patient's skin lesions. (A) A blister on the posterior neck; (B) Multiple blisters and erosion on the right arm; (C) Healed blisters and erosion on the arm without scarring.

**FIGURE 2:**
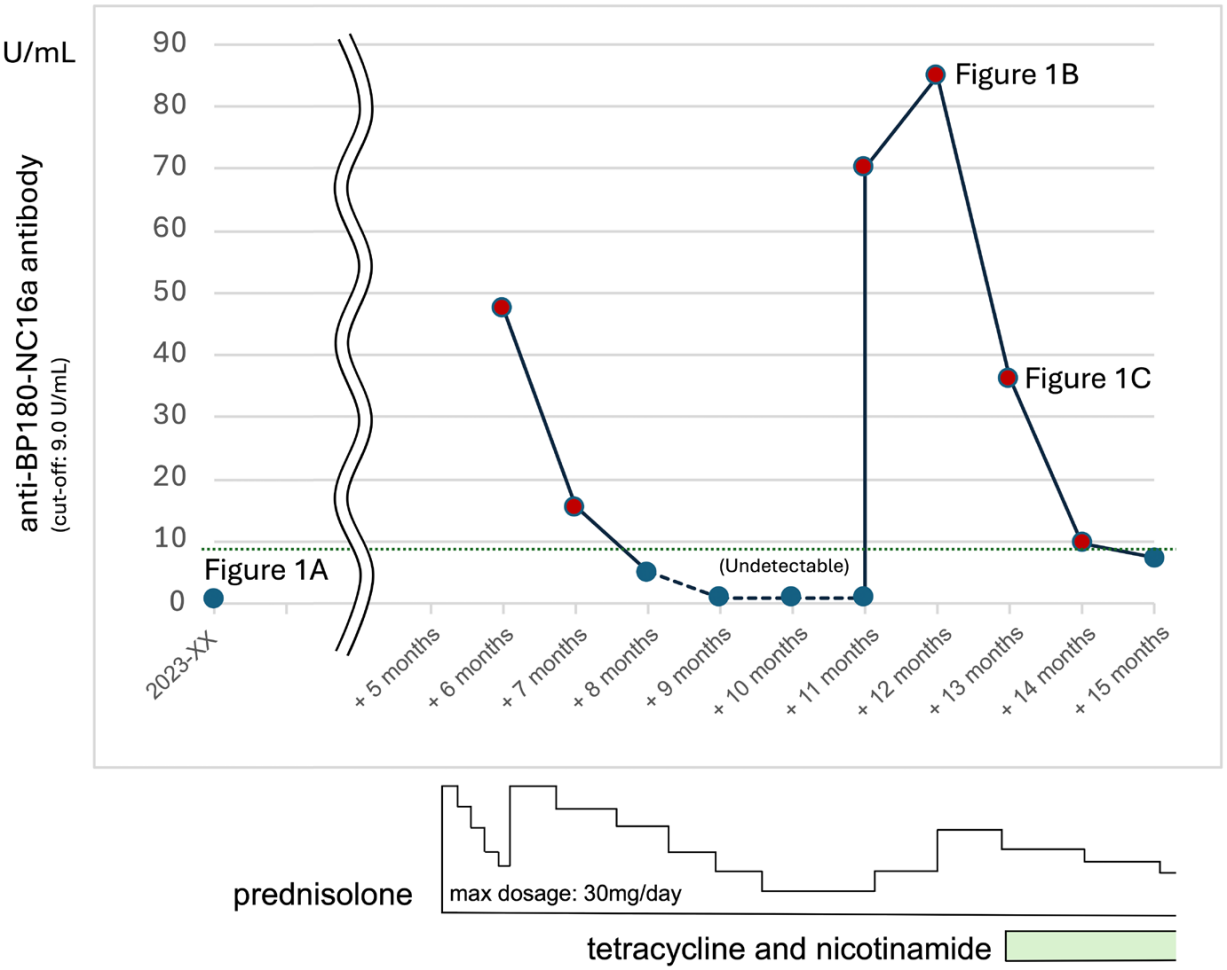
Course of anti‐BP180‐NC16a antibody levels and treatment. The graph illustrates the course of the anti‐BP180‐NC16a antibody levels (U/mL) over time, with corresponding treatment interventions.

Upon physical examination, tense blisters were observed on the posterior neck, negative for the Nikolsky sign, suggesting bullous pemphigoid (Figure 1A). However, the patient was negative for anti‐BP180‐NC16a antibody. Given the mild presentation, the home‐visiting physician opted for observation. Five months later, multiple tense blisters and erosion developed across the trunk and limbs, accompanied by erythema and limb edema. As a result of his limitations in ADLs, a hospital outpatient visit was not feasible. Instead, a dermatologist was consulted for a home visit. Drug eruption was initially suspected, leading to the discontinuation of antiepileptics and antipsychotics and the initiation of systemic corticosteroid therapy (prednisolone 30 mg) via the gastrostomy tube. Although the corticosteroid dosage was tapered weekly, the eruption did not resolve adequately, prompting the dermatologist to reconsider the diagnosis. Approximately 1 month after initiating corticosteroid therapy, a repeat test for the anti‐BP180‐NC16a antibody, as recommended by the dermatologist, returned positive (Figure 2). While a skin biopsy is typically performed in a hospital setting, the dermatologist performed the biopsy of a blister from the left lower leg at the patient's home. The histopathological examination revealed subepidermal blisters and lymphocytic infiltration in the upper dermis, confirming the diagnosis of bullous pemphigoid.

Since the patient's family wanted to avoid hospitalization, collaborative home‐based care was provided. The general practitioner managed the potential side effects of the corticosteroid, while the dermatologist adjusted its dosage. Home‐visiting nurses assisted the patient's caregiver with skincare and the application of topical treatments, including diflorasone diacetate (a high‐potency corticosteroid used as adjunctive therapy for moderate‐to‐severe bullous pemphigoid to enhance the effects of systemic corticosteroid therapy) and ozenoxacin lotion (an antibiotic for the suppression of localized bacterial infections). Meanwhile, home‐visiting pharmacists adjusted the forms of medication. Gradually, the erythema, blisters, and erosions subsided, and the anti‐BP180‐NC16a antibody levels decreased (Figure 2). While tapering the corticosteroid, the nurses noted the recurrence of erythema, tense blisters, and erosion on the limbs and trunk. The anti‐BP180‐NC16a antibody levels had risen again, prompting the dermatologist to adjust the corticosteroid dosage. The visiting nurses provided 14 consecutive days of care (except weekends), with each session lasting up to 90 minutes. Disease resolution was seen within 1 month (Figure 1B,C). Currently, the patient has achieved clinical remission, maintained on 10 mg/day of prednisolone, alongside tetracycline and nicotinamide. The anti‐BP180‐NC16a antibody levels became negative again.

## 3 DISCUSSION

In this case, the initial anti‐BP180‐NC16a antibody test was negative. However, the diagnosis of bullous pemphigoid was supported by the positive antibody levels on repeat testing and skin biopsy. The patient was successfully treated through home‐based care. This case report has two significant implications. First, in home care settings, repeated antibody testing may be considered if the disease severity increases, especially since the initial tests can yield false negatives. Second, bullous pemphigoid can be managed at home through collaborative practice.

No previous reports have discussed NC16A reactivity that developed later in the disease.^4^ In most patients, NC16A is recognized by the humoral immune system early in the disease, whereas epitope spreading to NC16A rarely occurs in patients without anti‐NC16A IgG.^4^ Our case suggests that reactivity to NC16A can emerge later, but the mechanism behind this delayed seroconversion remains unknown.^3^, ^4^ In a previous study, most patients with bullous pemphigoid who tested negative for anti‐BP180‐NC16a antibodies did not exhibit erythema and had relatively mild phenotypes.^3^ Therefore, the antibody levels may have lower sensitivity in patients presenting with blisters early in the disease, such as in this case. Thus, the antibody levels should be rechecked in patients who are initially negative but later develop erythema and worsening disease severity, especially when access to dermatologists is limited. Notably, the anti‐BP180 antibody levels correlate with the disease severity of bullous pemphigoid,^3^ allowing for disease monitoring and corticosteroid dosage adjustments based on antibody titers.

Skin biopsy is often recommended for cases of suspected bullous pemphigoid, but this may be challenging for patients who are unable to visit the hospital. Bullous pemphigoid is associated with neurological diseases such as stroke,^5^ and older adults, who are more susceptible to this condition, may face significant barriers to hospital care. General practitioners can perform skin biopsies in some countries, but this varies.^6^, ^7^ Skin biopsy is not a standard procedure in the Japanese training curriculum for general practice, making it uncommon among these doctors. Thus, interdisciplinary collaboration is crucial between general practitioners and dermatologists, particularly in home‐based care.

This patient was successfully managed at home through multidisciplinary collaboration, despite the typical approach involving hospitalization.^2^ A key component was the nursing visits lasting 90 minutes per session, which included cleaning the erosions and applying ointment. Japanese medical insurance allows for up to 14 consecutive days of home care visits, with each visit lasting up to 90 minutes under a physician's direction. The nurses also played a crucial role in the early detection and response to deteriorating conditions.^8^ Additionally, the pharmacist contributed by ensuring the safe adjustment of medications administered via the gastrostomy tube.^9^ Using their expertise, the pharmacist evaluated whether specific medications were compatible with administration through the tube (e.g., ensuring doxycycline could be dissolved in water). Considering factors such as the patient's family environment and preferences, as well as caregiver‐ or healthcare‐related issues (e.g., limited medical or nursing support),^10^ it may be feasible to manage severe bullous pemphigoid in a home‐based setting.

## 4 CONCLUSION

In bullous pemphigoid, the anti‐BP180‐NC16a antibody may exhibit a delayed seropositive conversion as the disease activity progresses. Reassessing antibody levels in cases of worsening disease severity is recommended since this could offer valuable insights for guiding ongoing management.

## AUTHOR CONTRIBUTIONS

**Hiromitsu Yamashita:** Writing – original draft. **Takayuki Ando:** Writing – review and editing. **Hirohisa Fujikawa:** Writing – review and editing. **Masayoshi Shiota:** Supervision; writing – review and editing.

## CONFLICT OF INTEREST STATEMENT

The authors declare that there are no conflicts of interest.

## ETHICS STATEMENT

Ethics approval statement: Ethics committee approval was not required as this is a single‐patient case report without experimental intervention.

Patient consent statement: Written informed consent was obtained from the patient and his wife for the publication of this case report and any accompanying images.

Clinical trial registration: None.

## Data Availability

The data supporting the findings of this case report are available within the article. Additional details can be provided by the corresponding author upon reasonable request while maintaining patient confidentiality.
